# Using *Plantago major* and *Plantago lanceolata* in environmental pollution research in an urban area of Southern Poland

**DOI:** 10.1007/s11356-019-05535-x

**Published:** 2019-06-14

**Authors:** Iryna Skrynetska, Jagna Karcz, Gabriela Barczyk, Marta Kandziora-Ciupa, Ryszard Ciepał, Aleksandra Nadgórska-Socha

**Affiliations:** 1 Department of Ecology, Faculty of Biology and Environmental Protection, University of Silesia, Bankowa 9, 40-007 Katowice, Poland; 2 Scanning Electron Microscopy Laboratory, Faculty of Biology and Environmental Protection, University of Silesia, Jagiellońska 28, 40-032 Katowice, Poland

**Keywords:** SEM-EDX, APTI, Potentially toxic metals (PTMs), *Plantago*, Soil pollution

## Abstract

The aim of this study was to perform a complex assessment of changes in the elements of an ecosystem that are caused by environmental pollution in industrial and urban biotopes. The study focused on three sites: a park, a road and the site of the metallurgical plant “Pokój” in the city of Ruda Śląska (Southern Poland), which are each under a different level of anthropogenic load. Soil and plant material samples (*Plantago major* and *Plantago lanceolata* leaves) were investigated by performing biochemical, ecophysiological and scanning electron microscopy (SEM) analyses. A significant difference was observed in all of the study samples. The content of Pb, Zn and Cd in the soil samples that had been collected at the site of the metallurgical plant exceeded the permitted limits (Cd > 4 mg kg^−1^, Pb > 100 mg kg^−1^, Zn > 300 mg kg^−1^). The content of Fe, Mn, Pb, Cd and Zn in the plant material was much higher in unwashed samples than in washed samples. The concentrations of potentially toxic elements (PTEs) were below the permitted level in the leaves of *Plantago lanceolata* for Cd (> 5 mg kg^−1^) and in the leaves of *Plantago major* for Zn (> 100 mg kg^−1^). The SEM observations revealed a significant decrease in the stomata pore length (SPL) in the *Plantago lanceolata* leaves that had been collected at the road site compared with the plants from the park site. The elemental content on the leaf surface was also determined using X-ray microanalysis. The total chlorophyll (Chl) content, ascorbic acid (AA), proline, guaiacol peroxidase (GPX) activity, pH, relative water content (RWC) and air pollution tolerance index (APTI) were evaluated. The APTI for the investigated species ranged from 5.6 to 7.4, which demonstrated that the studied plant species are sensitive to air pollutants.

**Electronic supplementary material:**

The online version of this article (10.1007/s11356-019-05535-x) contains supplementary material, which is available to authorized users.

## Introduction

Over the last several decades, the quality of the environment has undergone a significant deterioration, which was primarily due to rapid developments in industry as well as urbanisation. Environmental pollution has become a factor that is responsible for many negative effects on the health of fauna and flora as well as on the ecosystem as a whole because of potentially toxic metals that do not degrade and accumulate in the environment, most of which have long-term toxic effects on living organisms (Kardel et al. 2010; Remon et al. 2013; Muszyńska et al. 2018). However, the effects of this interaction on the function and structure of the elements of an urban ecosystem have not yet been adequately quantified and are poorly understood.

In flora, the epidermis is the first site of interaction with atmospheric pollution because pollutants first pass through the stomata of the epidermal tissues. The stomata, which regulate the flow of gases entering into or escaping out of leaves, are an excellent site to study the interaction between plants and their environment because they are the first to be affected by air pollution, which may cause changes in their morphology (Robinson et al. 1998; Kardel et al. 2010; Uka et al. 2017). There are many different biochemical and physiological mechanisms that help plants adapt to pollutants, and their efficiency can be assessed by a number of parameters such as the total chlorophyll (Chl) content, ascorbic acid (AA) content, pH and relative water content (RWC). All of these indexes make up the so-called air pollution tolerance index (APTI). The value of the APTI defines a plant’s tolerance to pollution because these parameters determine a plant’s adaptation to the environment and thus predetermine the sensitivity or resistance of a species to pollution (Lakshmi et al. 2008; Prajapati and Tripathi 2008). Additionally, a biochemical assessment of variations in metabolites could be helpful in defining the tolerance of a species. Proline accumulation is regarded as an indicator of heavy metal stress and enzymatic antioxidant components such as GPX may be used as an indicator of environmental stress for an ecosystem (Kandziora-Ciupa et al. 2017; Nadgórska-Socha et al. 2017).

The aim of this study was to perform a complex assessment of changes in the elements of an ecosystem that are caused by environmental pollution in industrial and urban biotopes. Two ruderal species, *Plantago major* and *Plantago lanceolata*, were selected for this study. The *Plantago* species has been used as a traditional medicinal plant in many parts of the world for centuries (Abd El-Gawad et al. 2015; Gomes de Andrade et al. 2018). *Plantago lanceolata* and *Plantago major* are easy to recognize and are very common in urban environments and in the countryside. Previous studies have indicated that the *Plantago major* and *Plantago lanceolata* species contain significant levels of trace elements (Tinkov et al. 2016; Nadgórska-Socha et al. 2017; Skrynetska et al. 2018).

The objective of this study was to perform a comparative analysis of selected ecophysiological and biochemical parameters and to determine the metal concentrations in soils and plants in samples that had been collected from three areas with different levels of the anthropogenic load. The data obtained enabled us to observe any differences in the morphology and physiological parameters, to analyse the air pollution tolerance indexes and to assess the potential use of the tested species as a bioindicator in an urban biotope. The tolerance of these plants to metal toxicity was established in order to determine their possible application in soil phytostabilisation and revegetation in industrial areas that have been contaminated with potentially toxic metals (Serbula et al. 2012; Nadgórska-Socha et al. 2013; Romeh et al. 2016). These results may be useful in evaluating the adaptive properties of these plants to harsh environmental conditions as well as their use in ecological risk assessment (Djingova et al. 2004; Przedpełska and Wierzbicka 2007; Słomka et al. 2008).

The following hypotheses were evaluated:

- Metal pollution contributes to changes in the ecophysiological and morphological properties of selected species within polluted sites compared with plants from a non-contaminated area.
- *Plantago* species may be useful biological indicators for industrialised urban areas.

## Material and methods

### Study area

The investigated areas represented a variety of habitats (green belts, squares, lawns and park) with ruderal and invasive species such as *Robinia pseudoacacia*, *Solidago canadensis* and *Reynoutria japonica*. Ruderal species were represented by *Taraxacum officinale*, *Achillea millefolium*, *Bellis perennis*, *Trifolium repens*, *Poa annua*, *Medicago lupulina* and others.

**Fig. 1:**
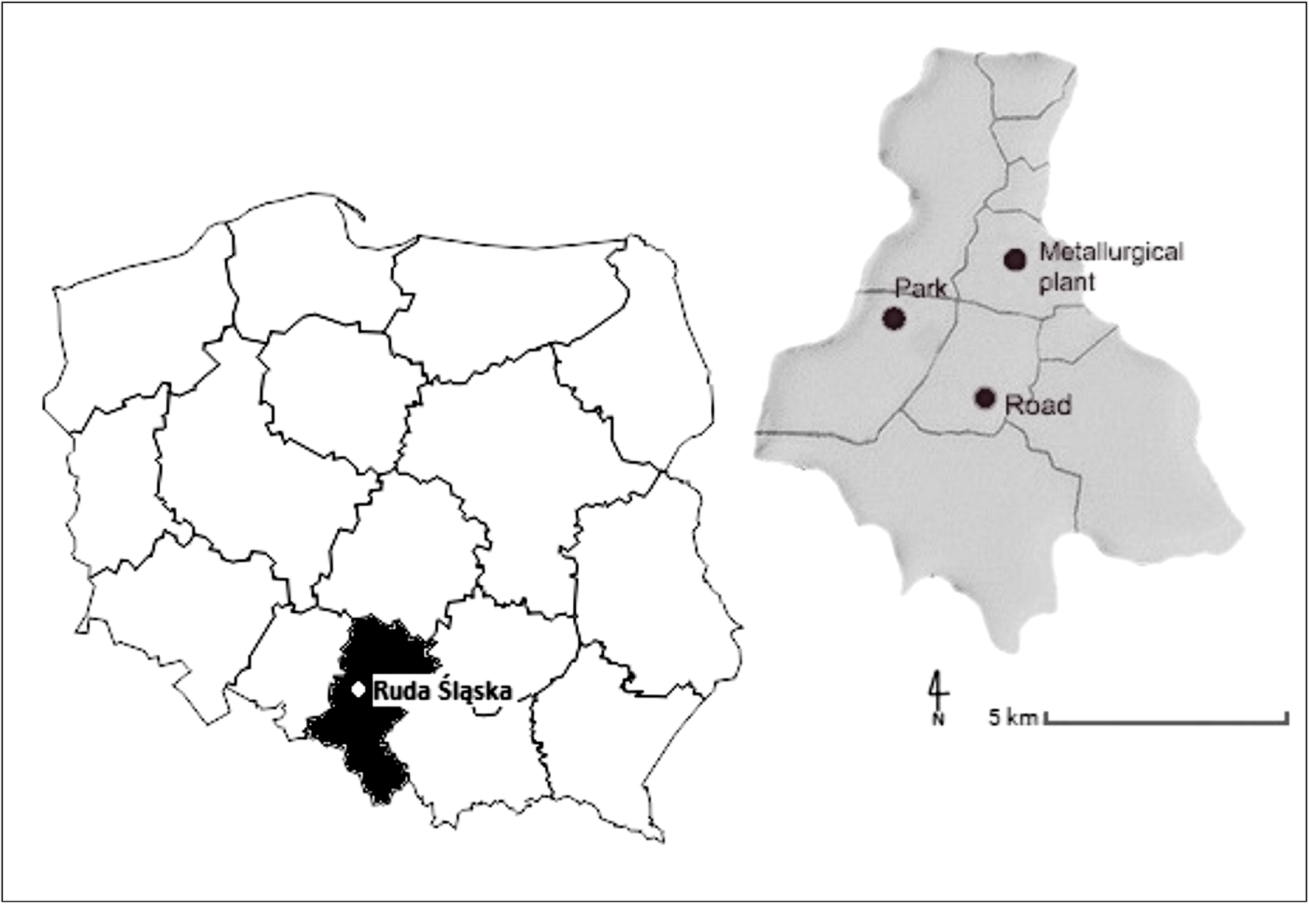
Location of the study sites in Ruda Śląska: the road (expressway A4); the site of the metallurgical plant “Pokój” and “Strzelnica” Park

The study sites were located in the city of Ruda Śląska (Upper Silesian Industrial District, Southern Poland). For the study, three locations were selected: a road (50°15′17.9″ N, 18°51′17.1″ E), a metallurgical plant (50°17′30.5″ N, 18°52′25.7″ E) and a park (50°16′28.4″ N, 18°50′12.8″ E) (Fig. 1).

The “Strzelnica” Park is a recreational and leisure area and is considered to be a potentially “clean” area. The road is the intersection of the A4 expressway and the provincial road 925, which has intensive road traffic. The metallurgical plant “Pokój,” where steel products are produced and distributed, is a site with a high level of environmental pollution.

### Soil and plant material collection

Soil samples were taken from the top layer at 0–10 cm depth from five locations at each site. The samples were collected during the vegetation season in late June and early July 2016. The soil and plant material samples were collected in five replicates at each site (i.e. a total of 15 soil samples and 15 plant material samples).

Plant materials from herbaceous lawns were selected: greater plantain (*Plantago major*) and narrow leaf plantain (*Plantago lanceolata*), which are species of *Plantago*, family *Plantaginaceae*. These two ruderal species are common and widespread and are also well known as good biological indicators (Kurteva 2009, Nadgórska-Socha et al. 2013, Romeh et al. 2016, Giacomino et al. 2016). The plant material for the biochemical analysis was frozen immediately after collection and kept frozen until the analysis.

### Scanning electron microscopy with energy-dispersive X-ray spectroscopy analysis

SEM was used to investigate the micromorphology of the leaf surfaces and stomata size. Leaves from plants of about the same age were taken randomly. Small pieces of fresh leaves near the central nerve (0.5 × 1 cm^2^) were cut from the same area of the leaf lamina, fixed in 3% glutaraldehyde in a 0.1 M sodium phosphate buffer, washed three times with the same buffer and then dehydrated with ethanol. In the next step, the samples were critical-point dried in a Pelco CPD2 apparatus (Ted Pella Inc., Redding, CA, USA) and then mounted on aluminium stubs with double-sided adhesive carbon tape and at lastly sputter coated in a Pelco SC-6 sputter coater (Ted Pella Inc.) with a 20 nm layer of gold in order to improve the electrical conductivity properties of the samples. All specimens were imaged using a field emission scanning electron microscope (Hitachi SU8010 FESEM; Hitachi High-Technologies Corporation, Tokyo, Japan), which was equipped with a secondary electron detector (ESD). The working conditions were 5 kV or 15 kV accelerating voltages, a working distance (WD) ranging from 8 to 25 mm.

Energy-dispersive X-ray microanalysis (EDX) with a detection limit of 0.1% of weight and beam penetration of 2–5 μm was used to identify the elemental content on the leaf surface using dry plant material that had not been fixed in GA. The parts of the leaves were mounted on aluminium stubs with double-sided adhesive carbon tape and sputter coated with gold. The specimens were examined using a field emission scanning electron microscope (FESEM) and a Thermo Scientific NORAN System 7 energy-dispersive spectrometer (Thermo Fisher Scientific, Madison, WI, USA). Background and element specific peak spectra were analysed with NSS 3 X-ray Microanalysis software (Thermo Fisher Scientific). SEM mode microanalysis was carried out at a 15-kV acceleration and the acquisition time was set to 60 s. Analyses were performed at × 500–× 1100 magnifications on 1–5 points of ten randomly selected pieces of the leaves from all of the investigated sites.

### Metal content analysis

The metal content of the soil was determined as pseudo-total HNO_3_ extractable fraction as was described in detail by Zheljazkov and Nielsen (1996). Additionally, metals were also extracted from the soil samples with 0.01 M CaCl_2_ (potentially available elements) according to Wójcik et al. (2014). The metal content was measured in the filtered extracts using atomic absorption spectroscopy (Thermo Fisher Scientific iCE 3500).

Soil pH was determined using a standard method (Ostrowska et al. 1991) using a 1:2.5 soil to water ratio. Organic matter content (expressed in %) was estimated following the method of Ostrowska et al. (1991).

The content of trace elements in the plants was measured using atomic absorption spectrometry (Thermo Fisher Scientific iCE 3500). The plant samples were divided into two groups and analysed as “washed” and “unwashed” samples. The “washed” plants were thoroughly washed with distilled water in an ultrasonic bath (ULTRON, Olsztyn, Poland) for 10 min at 20 °C to remove any dust deposits and then rinsed twice with distilled water. The plant samples were dried at 105 °C and then ground in a stainless steel mill; then, 0.25 g of the samples was wet digested in concentrated HNO_3_ at a maximum of 120 °C and finally diluted to 25 ml with deionised water (Lin et al. 2008).

### Biochemical analyses

Root viability was determined by measuring the GPX activity according to Fang and Kao (2000). Proline accumulation in the leaves was determined using the acid ninhydrin method (Bates et al. 1973). The RWC for the plant samples was determined according to Pathak et al. (2011). The pH value of the leaves was determined using a pH meter after homogenising 5 g f.w. of the leaves in 10 ml deionised water (Nadgórska-Socha et al. 2017). The content of total chlorophyll in the samples was quantitatively determined (Prajapati and Tripathi 2008) in accordance with Arnon (1949). The quantitative determination of ascorbic acid was performed according to Keller and Schwanger (1977) and as described in detail in Nadgórska-Socha et al. (2016).

The calculation of the air pollution tolerance index enables the degree of a plant’s tolerance to environmental pollution to be defined. The APTI was calculated according to Prajapati and Tripathi’s (2008) formula:

$$ \mathrm{APTI}=\frac{\mathrm{A}\times \left(\mathrm{T}+\mathrm{P}\right)+\mathrm{R}}{10} $$

where A is the ascorbic acid content (mg g^−1^ fresh weight); T is the total leaf chlorophyll content (mg g^−1^ fresh weight); P is the pH of leaf extract; R is the relative water content (%). According to Singh and Rao (1983), plants with APTI < 10 are sensitive; 10 < APTI < 16 are medium sensitive and APTI > 17 are resistant to air pollution.

Extra material about the methodology that was used is included in the supplementary material (Online Resource 1).

### Statistical analyses

All of the statistical calculations were performed using Statistica version 13 (StatSoft Inc., Tulsa, OK, USA). The observations were replicated five times for each parameter. The mean standard error was also calculated. Significant statistical differences were estimated using Tukey’s test. The Pearson coefficient of correlation for assessing estimated parameters was also calculated. Analysis of variance (ANOVA) helped to determine the variables that were significantly different among the soil and plant materials.

## Results and discussion

### Soil analysis

Soil pollution, particularly due to potentially toxic metal contamination, has been widely investigated by researchers around the world as one of the major environmental problems that can affect plant productivity, the environment and human health (Ross 1994; Alloway 1997; Kabata-Pendias and Pendias 2001; Kandziora-Ciupa et al. 2016). Previous soil metal accumulation researches that have been conducted in the urban areas of Upper Silesia (Miasteczko Śląskie, Chorzów, Piekary Śląskie, Sosnowiec, Dąbrowa Górnicza) have also reported excessive concentrations of Pb, Cd and Zn especially in soil samples that had been collected from areas near metallurgical plants (Nadgórska-Socha et al. 2013, 2016; Kandziora-Ciupa et al. 2013; Dziubanek et al. 2015; Skrynetska et al. 2018). Most of these studies were based on the fractions of the extracted elements. According to Zheljazkov et al. (2008), while the pseudo-total or HNO_3_ extractable soil metal concentrations are important, the phyto-available forms of specific metals in the soil are the ones to which plant roots are actually exposed. Amoakwah et al. (2013) noted that CaCl_2_ mobilises both Cd and Zn because of the combined effect of complexation by the chloride anion and cation exchange.

Taking into consideration both points, in our study, we elected to use both methods of metal extraction. According to the Regulation by the Minister of Environment (2002), the metal concentrations in the soil pseudo-total fraction, particularly cadmium, lead and zinc, exceeded the permissible concentrations at the site of the metallurgical plant site (4 mg kg^−1^, 100 mg kg^−1^ and 300 mg kg^−1^, respectively). The potentially toxic elements are usually extracted to a greater extent using HNO_3_ extraction rather than CaCl_2_ extraction, which was confirmed by our study. In most cases, the potentially bioavailable toxic metal content was below 1% of the estimated content of the elements in the soil fraction that had been extracted using HNO_3_. In the CaCl_2_ extracted concentrations, the highest content of Mn, Zn and Cd was recorded at the park, which may be connected with low pH. By contrast, the CaCl_2_ extracted fraction had a comparable level of lead with an average of 0.5 mg kg^−1^ and iron content with an average 0.4 mg kg^−1^ for all of the investigated sites (Table 1). Our results are similar to a study in the nearby Miasteczko Śląskie, Poland (Nadgórska-Socha et al. 2016). According to Meers et al. (2007), the 0.01 M CaCl_2_ extraction procedure proved to be the most versatile because it provided a good indication of phytoavailability.

**Table 1:** Analysis of the soil samples.

Stand	Extraction	Mn (mg kg^−1^)	Fe (mg kg^−1^)	Pb (mg kg^−1^)	Cd (mg kg^−1^)	Zn (mg kg^−1^)	Organic matter (%)	pH
Road	HNO_3_	236 ± 68^a^	6198 ± 1124^a^	34.7 ± 2.4^a^	0.7 ± 0.1^a^	133 ± 5^a^	6.0 ± 0.1^a^	7.0 ± 0.3^b^
	CaCl_2_	0.8 ± 0.2^a^	0.3 ± 0.1^a^	0.5 ± 0.1^a^	0.1 ± 0.0^a^	0.1 ± 0.0^a^		
Park	HNO_3_	820 ± 41^b^	8765 ± 342^bc^	51.2 ± 6.5^a^	2.1 ± 0.1^b^	237 ± 17^a^	8.1 ± 0.1^b^	5.5 ± 0.1^a^
	CaCl_2_	27 ± 4^ab^	0.4 ± 0.1^a^	0.5 ± 0.1^a^	0.5 ± 0.0^b^	15 ± 1^b^		
Metallurgical plant	HNO_3_	596 ± 40^a^	42,320 ± 1678^c^	693 ± 63^b^	7.2 ± 0.6^c^	2222 ± 228^b^	13.0 ± 0.1^bc^	7.2 ± 0.6^c^
	CaCl_2_	0.9 ± 0.1^a^	0.4 ± 0.1^a^	0.6 ± 0.1^a^	0.1 ± 0.0^a^	0.5 ± 0.1^a^		

Data is expressed as the mean ± SD. The different letters denote significant differences between specific metal concentrations in the fraction that had been extracted with HNO_3_ and CaCl_2_, organic matter content, and pH (*p* < 0.05)

The results that were obtained from the investigated locations provide clear information about the impact of pollution on a natural environment that is under pressure from industrialisation and urbanisation. Soils in a city are characterised by a high level of acidity and show a high level of mechanical damage as a result of human activity. Despite this, in our study, the pH of the surface soils at the road and the site of the metallurgical plant were nearly neutral, thus confirming the efficiency of the revitalisation programmes that began in 2015 (The Local Revitalisation Programme of the City of Ruda Śląska until 2030 (2015)). The study of the selected sites showed that the average level of organic matter was 9%. The lowest content was found at the road (Table 1).

### Analysis of plant material

#### SEM observation

Accumulation of particles on surface of leaves depends on physico-chemical nature of the particulates and the characteristics of the contact surface (Bussotti et al. 1995; Liang et al. 2017). The interaction between plants and the atmosphere occurs mainly via the stomata and therefore can be considered to be an air quality indicator. A study of the stomatal characteristics is an inexpensive and easy way to obtain relevant results (Kardel et al. 2010).

**Fig. 2:**
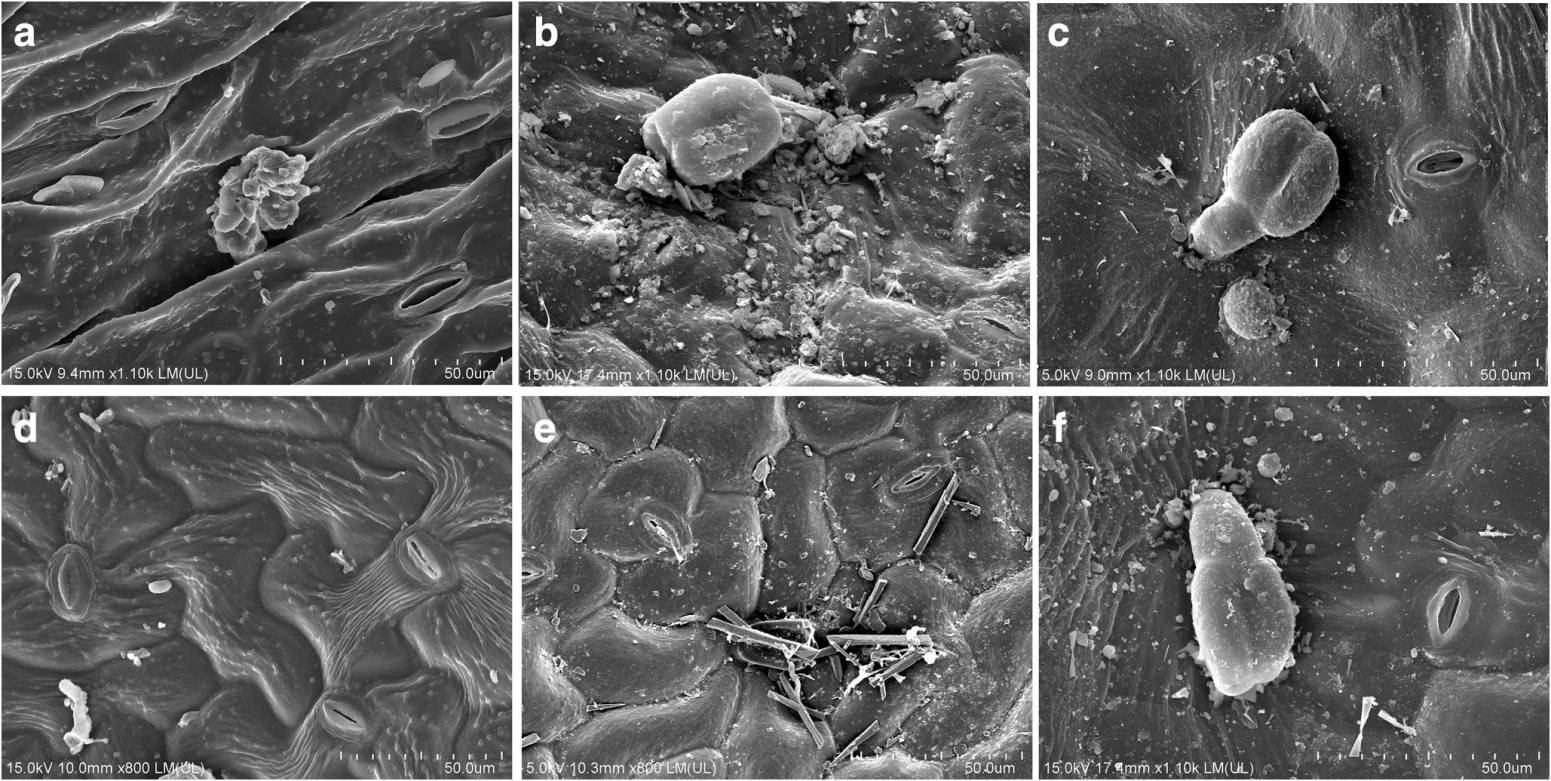
Representative SEM images of the adaxial surfaces of the leaves. **a***Plantago lanceolata* at the park. **b***Plantago lanceolata* at the road. **c***Plantago lanceolata* at the site of the metallurgical plant. **d***Plantago major* at the park. **e***Plantago major* at the road. **f***Plantago major* at the site of the metallurgical plant

A preliminary examination of the leaves was performed using light microscopy. *Plantago major* leaves have a blunt apex, 3–9 nerves, are sometimes slightly serrated, a naked or slightly hairy surface and a round shape. The leaves of *Plantago lanceolata* have a lanceolata or elliptic shape. Its leaf blade is usually full and rarely has a few serrations. In both of the investigated species, the abaxial surface of the leaves is lighter than the adaxial surface. No epicuticular waxes were present on the surfaces of the leaves. Fine deposits with irregular shapes and of different sizes were seen on the surfaces of the leaves in a polluted environment (the area near the road and the site of the metallurgical plant) (Fig. 2b, c, e, and f). At the road, the stomata were mostly closed and blocked by dust (Fig. 2b, e). Single trichomes were rarely observed on surfaces of the leaves from all of the investigated sites (Fig. 2b, c, and f).

Amphistomatous leaves and stomata occurred on both sides of the leaves in all of the *Plantago lanceolata* and *Plantago major* plants that were observed. The study of the micromorphology and anatomy of the *Plantago lanceolata* leaves using SEM revealed differences in the SPL in the area that has heavy traffic and near the site of the metallurgical plant compared with the park. The highest SPL values were found in the *Plantago lanceolata* (24.04 ± 1.26 μm) leaves at the park. Despite the fact that the SPL values in the leaves of *Plantago major* were much lower at the park (16.5 ± 0.75 μm), the lowest values for *Plantago lanceolata* (13.58 ± 0.95 μm) and for *Plantago major* (14.53 ± 0.65 μm) were recorded at the road. At the site of the metallurgical plant, the average SPL was 16.4 ± 0.91 μm and 18.23 ± 0.6 μm for *Plantago lanceolata* and *Plantago major*, respectively. The leaves of *Plantago major* had a comparable SPL at all of the investigated sites. A strong positive correlation was observed between the SPL and RWC, total chlorophyll content and APTI (*r*^2^ = 0.7, *r*^2^ = 0.55 and *r*^2^ = 0.7, respectively) and a negative correlation was observed between the SPL and ascorbic acid and proline content (*r*^2^ = − 0.58 and *r*^2^ = − 0.81, respectively). No correlation was observed between the SPL and metal content in either the washed or unwashed plant samples.

Wagoner (1975) reported no differences in the size of the stomata between polluted and unpolluted sites. Alves et al. (2008) described that an increase in stomatal density together with a decrease in stomatal size leads to an optimal adjustment for the control of gas exchange and the entrance of pollutants through the stomata. Moreover, Kardel et al. (2010) noticed a decrease in both the adaxial and abaxial stomata sizes in the leaves of *Plantago lanceolata* that acts as a mechanism for adapting to pollution stress in unsuitable habitats. The formation of smaller stomata in the leaf epidermis of trees was also recorded in Lublin, Poland (Chwil et al. 2015).

#### X-ray microanalysis

**Fig. 3:**
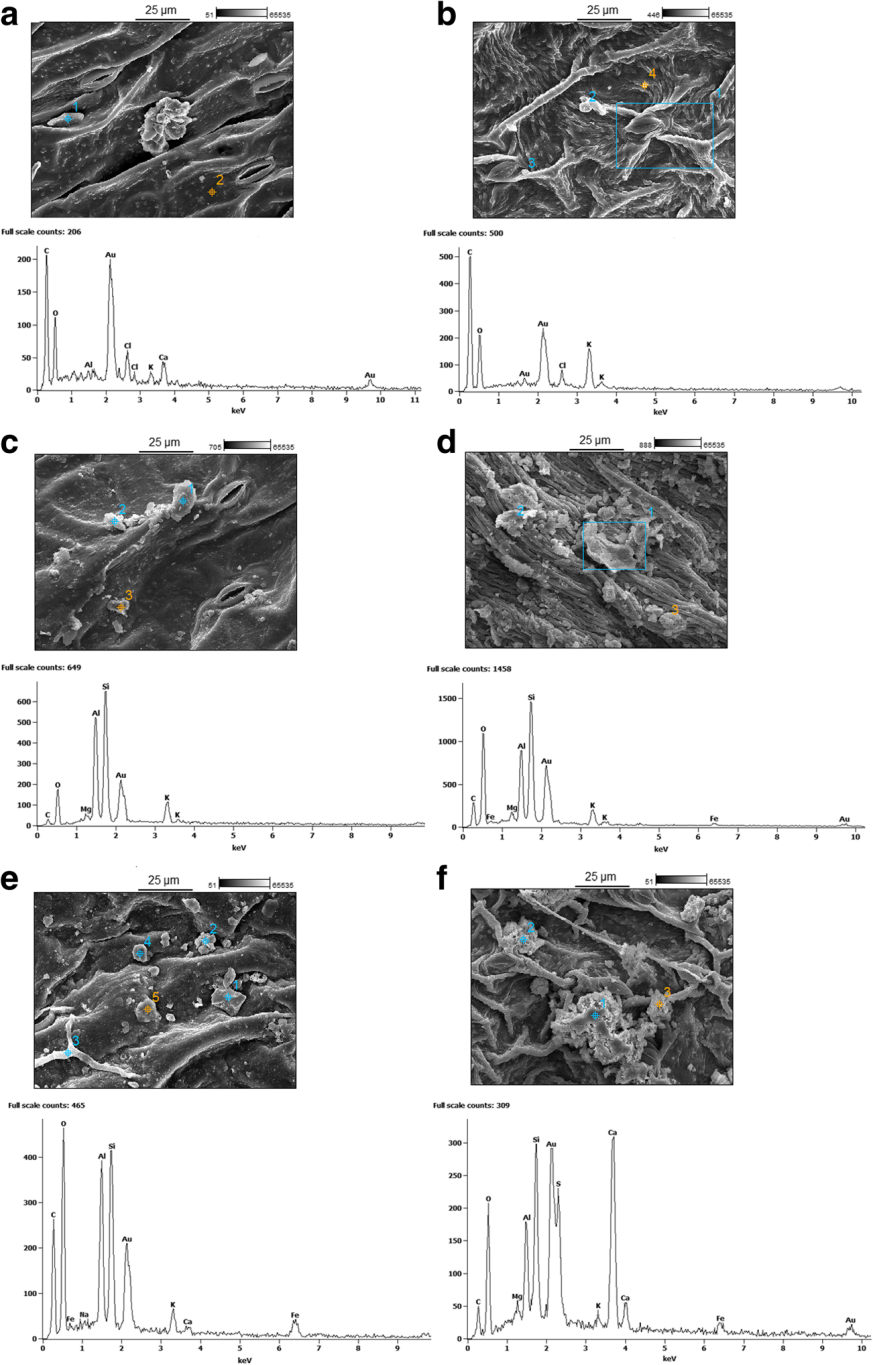
Selected SEM-EDX images of *Plantago* leaf samples with the chemical compositions of the most frequently identified elements on the adaxial surfaces. **a**, **b** The park. **c**, **d** The road. **e**, **f** The site of the metallurgical plant

Quantitative EDX analysis only provided information on the distribution of the elements and was not sensitive in depicting low concentrations of the elements (below the detection limit (> 0.1% weight)). We also analysed the elemental composition of the particles on the adaxial leaf surfaces. X-ray microanalysis revealed the presence of Si, Fe, S, Na, Ca, Mg, Cl, O, K and Al over the entire leaf surface sections that were examined. The results are presented as the averages of the spectra that were obtained at the study sites (Fig. 3). The gold (Au) signals can be considered to have originated from the sputter coating.

The element content on the adaxial leaf surfaces from the park was mainly represented by O, C, K, Cl, Ca and Mg (Fig. 3a, b), while at the road and the site of the metallurgical plant, additional elements were present (Na, Al, Si and Fe) (Fig. 3c–f). The highest peaks of Si and Al were recorded at the road, and a higher Fe content was recorded at the site of the metallurgical plant, along with the presence of Mn and fungal spores (not shown).

Almost the same elemental content was recorded on the surfaces in *Bignoniaceae* family leaves that had been collected from different areas of the Pune District, India (Kedar et al. 2018). In research conducted by Weerakkody et al. (2018) near a busy road, the amounts of C and O, in addition to Fe and Cl, were considerably larger compared with the other elements in PM_10_, and Ca, K, Si, Mg and S were present in particles of various sizes distributed on the leaves of all investigated species. According to Weerakkody et al. (2018), a high content of C and O can also indicate the presence of carcinogenic polycyclic aromatic hydrocarbons, primarily from fuel exhausts and tyre wear. Trace amounts of Ca, Ba, Mn, K, Mg and Zn can also be present in vehicle exhausts bound to organic components (Lin et al. 2005; Sharma et al. 2013). In addition to the dust that originates from road traffic, PM_10_ containing Al, Ca, Na, Si, Cl, F and N can originate from soil dust (Maher et al. 2013; Weerakkody et al. 2018).

#### Analysis of the metal content in the plant material

According to other researchers, the foliar metal uptake is mainly due to the soil–root pathway in urban and industrial environments (Schreck et al. 2012; Dao et al. 2014; Kandziora-Ciupa et al. 2017). The concentrations of the elements (Fe, Mn, Pb, Cd, Zn) were investigated in both washed and unwashed leaves of *Plantago major* and *Plantago lanceolata.* Higher metal concentrations were found in the unwashed samples, which was predictable as the dust on the surfaces of leaves can also contain metals (Maher et al. 2013; Weerakkody et al. 2018). In the washed plant material, the highest accumulation of Mn, Fe and Pb was recorded in the leaves of *Plantago lanceolata* at the site of the metallurgical plant (Table 2).

**Table 2:** Analysis of the metal content in *Plantago lanceolata* (Pl) and *Plantago major* (Pm)

		Mn (mg kg^−1^)		Fe (mg kg^−1^)		Pb (mg kg^−1^)		Cd (mg kg^−1^)		Zn (mg kg^−1^)	
		Washed	Unwashed	Washed	Unwashed	Washed	Unwashed	Washed	Unwashed	Washed	Unwashed
Road	Pl	14 ± 1^a^	41 ± 4^a^	76 ± 3^a^	408 ± 24^a^	0.4 ± 0.1^a^	4.0 ± 0.2^a^	7.9 ± 0.2^de^	10.0 ± 0.3^bc^	16.1 ± 3.2^a^	27.6 ± 2.9^a^
	Pm	20 ± 1^a^	25 ± 3^a^	90 ± 3^a^	308 ± 34^a^	0.9 ± 0.1^d^	4.7 ± 0.2^a^	0.1 ± 0.0^a^	0.5 ± 0.0^a^	25.3 ± 2.1^ab^	33 ± 3^a^
Park	Pl	18 ± 2^a^	46 ± 2^a^	51 ± 4^a^	443 ± 9^a^	0.4 ± 0.0^abc^	5.1 ± 0.1^a^	8.4 ± 0.1^f^	10.4 ± 0.3^bc^	38.6 ± 3.8^b^	70.8 ± 8.9^a^
	Pm	61 ± 3^b^	84 ± 4^a^	92 ± 8^a^	410 ± 35^a^	1.1 ± 0.0^c^	3.3 ± 0.1^a^	1.6 ± 0.1^c^	12.2 ± 0.5^c^	130 ± 6^d^	138 ± 9^a^
Metallurgical plant	Pl	133 ± 6^d^	307 ± 31^b^	391 ± 46^b^	1667 ± 110^b^	9.0 ± 0.3^e^	26 ± 3^b^	8.0 ± 0.1^e^	9.9 ± 0.4^bc^	73.8 ± 5.2^c^	165 ± 2^b^
	Pm	93 ± 8^c^	792 ± 4^c^	314 ± 17^b^	2830 ± 139^c^	0.6 ± 0.0^b^	27 ± 1^b^	0.8 ± 0.1^b^	10.2 ± 0.6^c^	27.8 ± 1.3^ab^	148 ± 7^a^

Data are expressed as the mean ± SD. The different letters denote significant differences between the metal concentrations in the various plants in the washed and unwashed samples (*p* < 0.05).

In the study area, the highest concentrations of Zn were found in the unwashed samples at the site of the metallurgical plant site for both of the species that were studied. The average was 61 mg kg^−1^ for *Plantago major* and 43 mg kg^−1^ for *Plantago lanceolata* in the washed samples, and the average was 106 mg kg^−1^ for *Plantago major* and 88 mg kg^−1^ for *Plantago lanceolata* in the unwashed samples (Table 2).

The iron content in the study area ranged from 51 to 391 mg kg^−1^ for the washed samples and from 308 to 2830 mg kg^−1^ for the unwashed samples. The highest concentrations were observed in both study species at the site of the metallurgical plant. The Pb concentration was a few times greater in the unwashed than in the washed plants. The highest manganese concentration was recorded in the leaves of *Plantago major* in the unwashed samples (792 mg kg^−1^) at the site of the metallurgical plant, which is eight times higher than the concentration in the washed samples (Table 2).

Potentially toxic concentrations of Cd (> 5 mg kg^−1^) were found in all of the samples of *Plantago lanceolata* leaves from the entire study area, according to the limits reported by Kabata-Pendias and Pendias (2001). Moreover, the permissible Cd content was exceeded in almost all of the unwashed samples, except for the leaves of *Plantago major* at the road (Table 2). Cd accumulation in edible plants has been found at significantly lower concentrations, i.e. 0.8 to 0.1 mg kg^−1^ (Dziubanek et al. 2015) compared with our results. A field study conducted by Nadgórska-Socha et al. (2017) also reported a lower metal content in *Taraxacum officinale*, *Plantago lanceolata*, *Betula pendula* and *Robinia pseudoacacia* leaves. Stafford et al. (2016) noted that the Cd accumulation in *Plantago lanceolata* ranged from 0.44 to 0.89 mg kg^−1^. In our study, the highest concentration of Zn in the washed samples, which exceeded the permissible concentration 100 mg kg^−1^ (Kabata-Pendias and Pendias 2001), was found in the leaves of *Plantago major* at the park site. By contrast, the study conducted by Kurteva (2009) recorded a higher Zn accumulation in the leaves of *Plantago lanceolata.* Our investigation of the accumulation of potentially toxic metals found much higher metal concentrations in the soil and leaves of *Plantago major* compared with the study in Cluj-Napoca, Romania, of Levei et al. (2018).

After averaging the data that was obtained in the measurements, the concentrations of the elements in the washed samples can be ranked in the following descending order for *Plantago lanceolata*: Fe > Mn > Zn > Cd > Pb and for *Plantago major*: Fe > Zn > Mn > Pb > Cd as was also recorded for *Plantago major* in Sosnowiec (Skrynetska et al. 2018). The order of the concentrations in the unwashed samples for both *Plantago* species was the same: Fe > Mn > Zn > Pb > Cd. This fact supports the crucial point that the plant samples that are used in biomonitoring studies must be washed.

#### Analysis of the biochemical parameters

To estimate the state of the environment, in addition to the above-mentioned results, the total chlorophyll, proline, ascorbic acid, relative water content and leaf pH were determined. The GPX activity was also analysed (Table 3).

**Table 3:** Analysis of the biochemical parameters of *Plantago lanceolata* (Pl) and *Plantago major* (Pm)

		GPX (μg g^−1^ f.w.)	Proline (μmol g^−1^ f.w.)	RWC (%)	Chl (mg g^−1^ f.w.)	pH	AA (mg g^−1^ f.w.)	APTI
Road	Pl	348 ± 4^a^	9.8 ± 0.5^b^	60 ± 3^a^	0.13 ± 0.01^ab^	4.5 ± 0.1^a^	0.48 ± 0.02^a^	6.2 ± 0.1^b^
	Pm	357 ± 4^a^	11.2 ± 2.2^bc^	54 ± 4^a^	0.09 ± 0.01^a^	5.0 ± 0.2 ^b^	0.40 ± 0.08^a^	5.6 ± 0.6^a^
Park	Pl	572 ± 6^a^	6.9 ± 0.4^ab^	72 ± 4^a^	0.17 ± 0.02^b^	5.2 ± 0.1 ^bc^	0.22 ± 0.04^a^	7.4 ± 0.5^b^
	Pm	434 ± 5^a^	8.3 ± 1.4^b^	69 ± 3^a^	0.16 ± 0.01^b^	5.6 ± 0.1^c^	0.51 ± 0.07^a^	7.2 ± 0.4^b^
Metallurgical plant	Pl	581 ± 6^a^	8.8 ± 0.2^b^	68 ± 3^a^	0.09 ± 0.01^a^	5.3 ± 0.1^c^	0.42 ± 0.04^a^	7.0 ± 0.3^b^
	Pm	1254 ± 63^b^	3.8 ± 0.6^a^	70 ± 2^a^	0.18 ± 0.01^bc^	5.0 ± 0.1^bc^	0.46 ± 0.08^a^	7.2 ± 0.2^b^

Data are expressed as the mean ± SD. The different letters denote significant differences between the contents of AA, Chl, RWC, pH, GPX, and proline (*p* < 0.05)

Proline accumulation is considered to be a common physiological response of many plants to environmental stress factors (Verbruggen and Hermans 2008; Tantrey and Agnihotri 2010). Moreover, researchers have found a significant amount of proline in the reproductive parts of different plant species, which raises the possibility that the accumulation of this amino acid may also occur in non-stressed physiological conditions (Mattioli et al. 2009). Numerous studies have also noted a higher content of proline in samples from contaminated areas compared with potentially clean sites (Tantrey and Agnihotri 2010; Kumar et al. 2010; Kandziora-Ciupa et al. 2016; Kandziora-Ciupa et al. 2017). In our investigation, the highest proline content was recorded at the road site for both of the study species (Table 3). The average proline contents for the *Plantago major* and *Plantago lanceolata* leaves were 7.8 μmol g^−1^ and 8.5 μmol g^−1^ f.w., respectively (Table 3). An increase in the proline level during environmental contamination was also found in *Philadelphus coronarius* leaves by Kafel et al. (2010) and confirmed in *Taraxacum officinale*, *Plantago lanceolata*, *Betula pendula* and *Robinia pseudoacacia* leaves because of urban environmental traffic contamination by Nadgórska-Socha et al. (2017). In a field study near the site of a smelter, a higher proline content was also recorded in the leaves of *Vaccinium murtillus* (Kandziora-Ciupa et al. 2017).

GPX activity is significant for plant growth and development. The activity of antioxidant enzymes changes under biotic and abiotic stress conditions and can be used as a potential indicator of metal toxicity and other stress factors (Verma and Dubey 2003; Doğanlar and Atmaca 2011; Kandziora-Ciupa et al. 2017). According to the obtained results, a higher level of GPX activity was recorded in the leaves of *Plantago major* at the site of the metallurgical plant (1254 tetra-guaiacol g^−1^ f.w.), while the lowest was recorded in the *Plantago lanceolata* leaves (348 tetra-guaiacol g^−1^ f.w.) at the road (Table 3). In our study, a lower GPX activity was recorded at the road and was positively correlated with the Fe and Mn content in the washed samples and with Pb, Zn, Fe and Mg in the unwashed samples. A similar dependence was also found in studies that were conducted by Kandziora-Ciupa et al. (2013, 2017). Many authors have reported increased GPX activity in response to elevated potentially toxic metal concentrations (Verma and Dubey 2003; Kafel et al. 2010; Doğanlar and Atmaca 2011; Nadgórska-Socha et al. 2013; Marchand et al. 2016).

Relative water content (RWC) is the level of water that is required in plants to maintain a physiological balance (Rai and Panda 2014). According to Krishnaveni (2013), RWC is one of ecophysiological indicators of environmental stress in plants. In our study, the average relative water content in the leaves of *Plantago major* was nearly 64% and was nearly 67% for *Plantago lanceolata*, which confirms that the selected plants are resistant to water stress. The lowest values for both species were observed at the road (Table 3).

The leaf pH, which is a common physiological parameter, is also suggested to be an indicator of plant stress (Krishnaveni et al. 2013; Husson et al. 2018). The pH of the extracts from the leaves in the study area ranged from 4.5 to 5.6. The lowest pH was recorded in both study species at the road. The average pH for the *Plantago major* leaves was 5.21, and it was 4.98 for *Plantago lanceolata* (Table 3). The values of the leaf pH that were obtained were lower for both species than the results of a field study in Sosnowiec (Poland) (Skrynetska et al. 2018). Krishnaveni et al. (2013) also recorded a decrease in the leaf pH values at polluted sites. By contrast, a laboratory study conducted by Cornelissen et al. (2011) reported that leaf pH is largely a species-specific trait, and therefore, the investigated species could maintain a leaf pH independently from the soil environment. Studies conducted by Sharma et al. (2013), Zhang et al. (2016) and Bharti et al. (2018) emphasised that a lower leaf pH is connected with the presence of SO_x_ and NO_x_ in the air. This fact suggested us to conclude that the leaf pH depends directly on air quality.

Studies on chlorophyll content are considered to be relevant as its level is connected with tolerance in contaminated environments (Pathak et al. 2011; Rai and Panda 2014; Ogunkunle et al. 2015; Nadgórska-Socha et al. 2017). We observed comparable results for *Plantago major* and *Plantago lanceolata*. The average contents in the leaves of *Plantago major* and *Plantago lanceolata* were 0.14 mg g^−1^ f.w. and 0.13 mg g^−1^ f.w., respectively (Table 3). Previous field studies have recorded a higher total chlorophyll content in the leaves of *Plantago major* and *Plantago lanceolata* in Sosnowiec, Poland (Skrynetska et al. 2018) and in the leaves of *Plantago lanceolata* in Dąbrowa Górnicza, Poland (Nadgórska-Socha et al. 2017)*.* The content of Chl can be affected by high temperature, drought, salt stress, light intensity, gaseous pollutants and potentially toxic metal contamination (Pandey et al. 2015; Zhang et al. 2016).

Another important indicator of physiological condition of a plant is the content of ascorbic acid (AA), which is a strong reducing agent that activates many defence mechanisms in plants, whereby increased ascorbic acid content enhances pollution tolerance (Pandey et al. 2015; Zhang et al. 2016; Nadgórska-Socha et al. 2017). Ascorbic acid is located mainly in the chloroplast and plays an important role in the synthesis of the cell walls, cell division and the processes that are associated with detoxification (Ogunkunle et al. 2015). In our study, the average AA content in the leaves of *Plantago major* was 0.46 mg g^−1^ f.w., while for *Plantago lanceolata*, it was 0.37 mg g^−1^ f.w. The lowest AA content was observed in the leaves of *Plantago lanceolate* at the park (Table 3). A much lower AA content was found in the leaves of *Plantago major* in a field study in Sosnowiec, Poland (Skrynetska et al. 2018). As was reported by Tripathi and Gautam (2007), an increase in the AA content in all plant species may be due to the increased rate of the production of reactive oxygen species. In a field study conducted by Nadgórska-Socha et al. (2016), a decreasing tendency was found in the leaves of *R. pseudoacacia*, and an increase in the AA content was found in the leaves of *M. album* at contaminated sites. Some studies have also reported a high concentration of AA at industrial sites (Agbaire and Esiefarienrhe 2009; Meerabai et al. 2012; Ogunkunle et al. 2015).

Calculating the air pollution tolerance index (APTI) enables the tolerance of plants to air pollution to be determined and the biochemical parameters that are responsible for resistance to environmental stress factors to be found. In our study, *Plantago major* and *Plantago lanceolata* had a narrow range of tolerance in the APTI index (5.6 to 7.4). It was found that the relative APTI average of *Plantago major* was 6.7 while it was 6.8 for *Plantago lanceolata*, thus indicating that both are sensitive to air pollution. The road site had the lowest APTI values for both of the study species (Table 3). According to the classification of Singh and Rao (1983), the investigated plants are species that are sensitive to air pollution. Low values of APTI were also noted in both contaminated and conventionally clean sites in Sosnowiec, Poland (Skrynetska et al. 2018). In Dąbrowa Górnicza, Poland, the APTI of *Plantago lanceolata* was higher (8.43–14.57), especially at non-contaminated sites compared with contaminated sites (Nadgórska-Socha et al. 2017). Another study in Southern Poland (Miasteczko Śląskie, Katowice, Jaworzno) using *Robinia pseudoacacia* and *Melandrium album* at potentially toxic metal-contaminated sites recorded a mean APTI value for all of the investigated sites at 9.4 for *R. pseudoacacia* and 8.7 for *M. album* (Nadgórska-Socha et al. 2016). Zhang et al. (2016) identified species that are tolerant to air pollution (*Magnolia denudata*, *Diospyros kaki*, *Ailanthus altissima*, *Fraxinus chinensis* and *Rosa chinensis*), which had been collected from two heavy traffic roadside sites and one suburban site in Beijing and recommended it to be planted at locations where there is heavy traffic. Bharti et al. 2018 estimated the APTI of 25 plant species that were growing at the Talkatora Industrial Area, India, and determined that *Polythalia longifolia* was the species that was most sensitive to air pollution.

A plant species with a higher APTI can be used in green belts and should be given priority for replantation in urban and industrial areas in order to reduce the effects of air pollution (Sisodia and Dutta 2016; Achakzai et al. 2017; Bharti et al. 2018). A plant species with a lower APTI can be recommended as a bioindicator and for environmental monitoring (Nadgórska-Socha et al. 2016, 2017; Bharti et al. 2018). The results that were obtained indicate that *Plantago major* and *Plantago lanceolata* species can be classified as being sensitive to air pollution and can be recommended for bioindicative research in urban and industrial areas.

A clear correlation was found between the pH value and the content of Mn and Zn (*r*^2^ = 0.5 and *r*^2^ = 0.87, respectively) and between the Chl and Pb content (*r*^2^ = − 0.85) in the washed plant material. Much stronger correlations were observed in the unwashed material. A correlation was found between the dehydrogenase activity and the content of Pb, Fe, Mn and Zn (*r*^2^ = 0.76; *r*^2^ = 0.92; *r*^2^ = 0.96 and *r*^2^ = 0.57, respectively). Significant positive correlations were found between the RWC and Cd and Zn concentrations (*r*^2^ = 0.81; *r*^2^ = 0.7, respectively), between the total chlorophyll and Cd content (*r*^2^ = 0.66) and between the pH value and the content of Zn (*r*^2^ = 0.72). Negative correlations were observed between proline content and the content of Pb, Fe, Zn and Cd in the unwashed samples (*r*^2^ = − 0.61; *r*^2^ = − 0.76; *r*^2^ = − 0.58 and *r*^2^ = − 0.56, respectively).

Although the *Plantago major* and *Plantago lanceolata* species that were investigated demonstrated different ecophysiological responses to environmental pollution, they can be recommended as unified bioindicators because of their wide dispersion in Europe, North America, and other regions of the world, e.g. South Africa (Kardel et al. 2010). The ability of this plant to accumulate metals can be also used in phytostabilisation and environmental risk assessment studies (Gucwa-Przepióra et al. 2016; Romeh et al. 2016). Moreover, it is important to continue this kind of research in order to determine plants with a tolerance or resistance to environmental pollution that can be used in developing green belts or to provide a low-cost and eco-friendly approach for reducing air pollution.

## Conclusions

The examinations of the leaves of *Plantago major* and *Plantago lanceolata* showed anatomical, biochemical and ecophysiological changes in the plant samples that had been collected from an industrialized urban area. Strong correlations were found between the SPL and the ecophysiological parameters (RWC, APTI, Chl, AA, proline content). The metal content also correlated with the biochemical and ecophysiological indexes to different degrees depending on the specific element.

The difference in metal concentrations between the washed and unwashed plant material is an essential distinction. The statistical analysis demonstrated the necessity of washing the plant material that is used in metal bioaccumulation studies because this factor affects the experimental accuracy.

According to the SEM-EDX results, a higher content of Al, Fe, Si and Mn was observed on the adaxial leaf surfaces at the road and metallurgical plant sites. During such an analysis, researchers should take into account the detection limit and depth of the beam penetration because tracking the trace element content in particles >2 μm is quite difficult. Therefore, for bioaccumulation studies, SEM-EDX analysis with an additional analysis of the metal concentration (AAS, ICP etc.) is recommended.

The results demonstrated that *Plantago major* had a higher tolerance ability to environmental pollution compared with *Plantago lanceolata* at the site of the metallurgical plant – an area with an extremely high metal content, which ensured its greater adaptation ability to stress factors*.* The calculation of the APTI index demonstrated that the plant species that were studied have a narrow range (5.6–7.4) and are sensitive to air pollutants, including potentially toxic metals, which suggests their usefulness as bioindicators of the environmental state.

## Electronic supplementary material

ESM 1 (PDF 420 kb)

## Abbreviations

AA: Ascorbic acid
APTI: Air pollution tolerance index
Chl: Total chlorophyll
GPX: Guaiacol peroxidase
RWC: Relative water content
SEM: Scanning electron microscopy
SEM-EDX: Scanning electron microscopy with energy-dispersive X-ray spectroscopy
SPL: Stomata pore length
