# Supplementary material for: Using Plantago major and Plantago lanceolata in environmental pollution research in an urban area of Southern Poland
Source: Environ Sci Pollut Res Int. 2019 Jun 14;26(23):23359–71. doi: 10.1007/s11356-019-05535-x (PMC6667404; doi:10.1007/s11356-019-05535-x)
Supplement: Supplementary file 1 — (PDF 420 kb) [file 11356_2019_5535_MOESM1_ESM.pdf]

Using *Plantago major* and *Plantago lanceolata* in environmental pollution research in an urban area of Southern Poland

Environmental Science and Pollution Research

Iryna Skrynetska[1], Jagna Karcz[2], Gabriela Barczyk[1], Marta Kandziora-Ciupa[1], Ryszard Ciepał[1], Aleksandra Nadgórska-Socha[1]

[1] Department of Ecology, Faculty of Biology and Environmental Protection, University of Silesia, Bankowa 9, Katowice, Poland, 40-007.

[2] Scanning Electron Microscopy Laboratory, Faculty of Biology and Environmental Protection, University of Silesia, Jagiellońska 28, Katowice, Poland, 40-032.

Corresponding author: Iryna Skrynetska. E-mail: i.skrynetska@gmail.com

Methodology

1. Soil and plant material collection

Soil samples were taken from the top layer of soil within the root zone at a depth of 0-10 cm (Dao et al. 2013, Elnazer et al. 2015, Kandziora-Ciupa et al. 2017). To reduce the heterogeneity, topsoil was sampled from five locations at each site (from 10 m × 10 m plots, from the central and four corner locations). The soil samples were transported in fabric bags to facilitate airflow and drying. The samples were sieved through a 2 mm mesh in order to remove stones and plant debris and mixed together to produce a representative sample.

Plant materials from perennial herbaceous perennials were selected: greater plantain (*Plantago major)* and narrow leaf plantain (*Plantago lanceolata)*, species of *Plantago*, family *Plantaginaceae*. These two species of ruderal are common and widespread, and well–known as good biological indicators (Kurteva 2009, Nadgórska-Socha et al. 2013, Romeh et al. 2015, Giacomino et al. 2016). Plant material for biochemical analysis was collected and immediately frozen until analysis.

The plant material and soil samples were collected during the vegetation season in late June and early July 2016. Attention was paid to the cleanliness and homogeneity of the samples during collection. Each

sample's date and place of harvest was described. From each site, the samples of soil and plant material were collected in five replicates.

2. SEM-EDX analysis

Scanning electron microscopy (SEM) was used to investigate the micromorphology of leaf surfaces and stomata sizes. Leaves from plants of about the same age were taken randomly. Small pieces of fresh leaves near the central nerve ($0.5 \times 1\ cm^2$) were cut from the same area of the leaf lamina, fixed in 3% glutaraldehyde (GA) in a 0.1 M sodium phosphate buffer, pH 7.2 for 24 h at room temperature, washed three times with the same buffer (15 min total), and then all the samples were dehydrated by ethanol (from 30% till 100% concentration, 10 min at each step). In the next step the samples were critical-point dried in Pelco CPD2 apparatus (Ted Pella Inc., Redding, CA, USA), and then mounted on an aluminum stubs with double-sided adhesive carbon tape, and afterwards sputter-coated in the Pelco SC-6 sputter coater (Ted Pella Inc.) with 20nm film of gold, to improve the electrically conducting properties of the sample surface. Finally, all specimens were imaged in a field emission scanning electron microscope (Hitachi SU8010 FESEM; Hitachi High-Technologies Corporation, Tokyo, Japan) with a secondary electron detector (ESD), at 5 kV and 15 kV accelerating voltages and working distance (WD) of 8 mm to 25 mm and acquisition time of 60 s.

Energy dispersive X-ray microanalysis (EDX) was applied to identify the elemental content on the leaf surface, with a detection limit of 0.1% of weight and beam penetration of 2-5 μm, using dry plant material not fixed in GA. The parts of the leaf were mounted on aluminum stubs with double–sided adhesive carbon tape and sputter-coated with gold. The specimens were examined by field emission scanning electron microscope (FESEM) and a Thermo Scientific NORAN System 7 energy dispersive spectrometer (Thermo Fisher Scientific Madison, WI, USA). Background and element specific peak spectra were analyzed with NSS 3 X-ray Microanalysis software (Thermo Fisher Scientific). SEM mode microanalysis was carried out at 15 kV acceleration and acquisition time was set to 60 s. Analyses were performed at 500–1100 magnification on 1–5 points of 10 randomly selected pieces of leaf from all the investigated sites. The SEM images and corresponding EDX spectra were taken for all specimens and one typical image and selected spectra of leaf surface are given in Figure 3.

3. Metal content analysis

Soil samples were air–dried, sieved through a 2 mm mesh, and stored at room temperature until analysis. The metal content of the soil was estimated in total and HNO_3–extractable fraction, as described in detail by Zheljazkov and Nielsen (1996). Soil samples (1 g) were soaked overnight in 10 ml of concentrated HNO_3 at room temperature, then decomposed further on an aluminium digestion block at 150 ºC for 8 hours, filtered through filter paper and then diluted to 50 ml with deionized water. Additionally, metals were extracted from soil samples with 0.01 M $CaCl_2$ (potentially bioavailable elements) according to Wójcik et al. (2014). For $CaCl_2$ extraction, 5 g of soil sample was mixed with 50 ml of 0.01 M $CaCl_2$ and mechanically shaken for 2 h, then filtered and stored at 0-4 ºC until analysis. The metal content was measured in the filtered extracts by atomic absorption spectroscopy (Thermo Fisher Scientific iCE 3500).

Soil pH was examined by standard method (Ostrowska et al., 1991) using a 1:2.5 soil to water ratio. Organic matter content (expressed in %) was estimated following the method by Ostrowska et al. (1991)

Metal content in the plants was measured by atomic absorption spectrometry. Plant samples were divided in two groups for the analysis of washed and unwashed plants. The "washed" plants were thoroughly washed with distilled water in an ultrasonic washing machine (ULTRON, Poland) for 10 min at 20 ºC to remove any dust deposits, and then rinsed twice with distilled water. Plant samples were dried at 105 °C, then ground in a stainless steel mill. Dry weight samples (0.25 g) were wet digested in concentrated HNO_3 at a maximum of 120 °C and then diluted to 25 ml with deionized water (Lin et al. 2008). Trace element contents (Cd, Pb, Zn, Fe, Mn) were measured by atomic absorption spectrometry (Thermo Fisher Scientific iCE 3500).

4. Biochemical analysis

Root viability was determined by measuring dehydrogenase activity according to Fang and Kao (2000). Plant material was homogenized in a 100 mM phosphate buffer at pH 6.8. Guaiacol peroxidase activity was measured in a reaction mixture (3 ml) containing 50 mM phosphate buffer (pH 5.8), 1.6 µl H_2O_2, 1.5 µl guaiacol and 0.2 ml enzyme extract, with absorbance measured at 470 nm with guaiacol as the substrate. The activity was calculated using the extinction coefficient (26 mM^{-1} cm^{-1}) for tetra-guaiacol and expressed in µmol tetra-guaiacol g^{-1} fresh weight min^{-1}.

Observation of proline accumulation in the leaves was carried out using an acid-ninhydrin method (Bates et al. 1973). Crushed plant material (0.5 g) was homogenized in 10 ml of sulfosalicylic acid (3 g per 100 ml). The reaction mixture with 2 ml of homogenate, 2 ml of ninhydrin acid and 2 ml of glacial acetic acid was incubated at 100 °C for 1 h. Then the reaction mixture was placed on ice and extracted with 4 ml of toluene. The

absorbance was read at 520 nm. The proline content was expressed in μmol proline g^{-1} fresh weight, calculated as described by Bates et al. (1973).

The relative leaf water contents (RWC) for plant samples were determined according to Pathak (2011) using the following formula:

$$RWC = [(FW-DW/TW-DW)] * 100 (\%)$$

FW – fresh weight (g);

TW – turgid weight (g);

DW – dry weight (g).

The pH value of the leaf was determined with a pH meter after homogenizing 5 g f.w. of leaves in 10 ml deionized water (Nadgórska-Socha et al. 2017). The content of total chlorophyll in the samples was quantitatively determined (Prajapati and Tripathi 2008) in accordance with Arnon (1949): 0.5 g of leaves was homogenized in 80% acetone solution, then decanted through a filter paper. The extraction was repeated and washed with acetone until discoloration of the solution. After that, the sample was supplemented with acetone to 50 ml and the absorbance measured at 645, 663 and 652 nm.

The quantitative determination of ascorbic acid was investigated according to Keller and Schwanger (1997) as previously described in detail in Nadgórska-Socha et al. (2016) using the following formula:

$$\text{Ascorbic acid} = \frac{(\text{Eo} - \text{Es} - \text{Et}) \times \text{V}}{\text{W} \times 100} \times 100 \ \ (\text{mg g}^{-1}\ \text{f.w.})$$

V – volume of the extract (ml);

W – weight of the leaf sample (g);

Eo, Es and Et – optical densities of blank sample, plant sample and sample with ascorbic acid.

Calculation of APTI (Air Pollution Tolerance Index). The APTI value allows us to define the degree of the plant's tolerance to environmental pollution. The most popular method is division into three classes/groups according to the level of sensitivity (Singh and Rao, 1983):

APTI < 10 = sensitive

10 < APTI < 16 = medium sensitive

APTI > 17 = resistant

For calculation of APTI, we used the following formula (Prajapati and Tripathi 2008):

$$\mathrm{APTI} = \frac{\mathrm{A} \times (\mathrm{T} + \mathrm{P}) + \mathrm{R}}{10}$$

A – ascorbic acid content (mg g^{-1} fresh weight);

T – total leaf chlorophyll content (mg g^{-1} fresh weight);

P – pH of leaf extract;

R – relative water content (%).

References

Arnon DI (1949) Copper enzymes in isolated chloroplast: polyphenol oxidase in Beta vulgaris. Plant Physiol 24:1-15.

Bates L, Waldren R, Teare D (1973) Rapid determination of free proline for water-stress studies. Plant Soil 39:205-207.

Dao L, Morrison L, Zhang H, Zhang C (2014) Influences of traffic on Pb, Cu and Zn concentrations in roadside soils of an urban park in Dublin, Ireland. Environ Geochem Health 36:333–343. doi: 10.1007/s10653-013-9553-8.

Elnazer A, Salman S, Seleem E, Abu El Ella E (2015) Assessment of Some Heavy Metals Pollution and Bioavailability in Roadside Soil of Alexandria-Marsa Matruh Highway, Egypt. Hindawi Publishing Corporation International Journal of Ecology 2015, Article ID 689420. http://dx.doi.org/10.1155/2015/689420.

Fang WC, Kao C (2000) Enhanced peroxidase activity in rice leaves in response to excess iron, copper and zinc. Plant Sci 158:71-76.

Giacomino A, Malandrino M, Colombo ML, Miaglia S, Maimone P, Blancato S, Conca E, Abollino O (2016) Metal Content in Dandelion (Taraxacum officinale) Leaves: Influence of Vehicular Traffic and Safety upon Consumption as Food. Journal of Chemistry, Volume 2016, http://dx.doi.org/10.1155/2016/9842987.

Kandziora-Ciupa M, Nadgórska-Socha A, Barczyk G, Ciepał R (2017) Bioaccumulation of heavy metals and ecophysiological responses to heavy metal stress in selected populations of Vaccinium myrtillus L. and Vaccinium vitis-idaea L. Ecotoxicology, vol. 26, iss. 7, 966-980.

Keller T, Schwanger H (1977) Air pollution and ascorbic acid. Eur J Forest Pathol 7:338-350.

Kurteva M (2009) Comparative study on Plantago major and P. lanceolata (Plantaginaceae) as bioindicators of the pollution in the region of the Asarel Copper Dressing Works. Phytologia Balcanica Sofia, 15 (2): 261-271.

Lin A, Zhang X, Zhu Y-G, Zhao F-J (2008) Arsenate induced toxicity: effects on antioxidative enzymes and DNA damage in Vicia faba. Environ Toxicol Chem 27:413-419. http://dx.doi.org/10.1897/07-266R.1

Nadgórska-Socha A, Ptasiński B, Kita A (2013) Heavy metal bioaccumulation and antioxidant responses in *Cardaminopsis arenosa* and *Plantago lanceolata* leaves from metalliferous and non-metalliferous sites. Ecotoxicology 22:1422-1434. https://doi.org/10.1007/s10646-013-1129-y

Nadgórska-Socha A, Kandziora-Ciupa M, Ciepal R, Barczyk G (2016) *Robinia pseudoacacia* and *Melandrium album* in trace elements biomonitoring and air pollution tolerance index study. Int. J. Environ. Sci. Technol. 13:1741-1752. https://doi.org/10.1007/s13762-016-1010-7.

Nadgórska-Socha A, Kandziora-Ciupa M, Trzęsicki M, Barczyk G (2017) Air pollution tolerance index and heavy metal bioaccumulation in selected plant species from urban biotopes. Chemosphere 183: 471-482. https://doi.org/10.1016/j.chemosphere.2017.05.128.

Ostrowska A, Gawliński S, Szczubiałka Z (1991) In: Method of analysis and estimate soil and plants property, Catalogue of the Environmental Protection Institute Warsaw pp 334-336 (in Polish).

Pathak V, Tripathi B, Mishra V (2011) Evaluation of Anticipated Performance Index of some tree species for green belt development to mitigate traffic generated noise. Urban Forestry & Urban Greening 10:61-66. http://doi.org/10.1016/j.ufug.2010.06.008.

Prajapati S, Tripathi B (2008) Seasonal variation of leaf dust accumulation and pigment content in plant species exposed to urban particulates pollution. J Environ Qual 37: 865-870. doi:10.2134/jeq2006.0511

Romeh A, Khamis M, Metwally S (2016) Potential of Plantago major L. for Phytoremediation of Lead-Contaminated Soil and Water. Water Air Soil Pollution 227: 9. https://doi.org/10.1007/s11270-015-2687-9.

Ross S (1994) Toxic metals in soil-plant systems. Chichester; New York: John Wiley & Sons.

Singh SK, Rao DN (1983) Evaluation of the plants for their tolerance to air pollution, Proceedings symposium on air pollution control held at IIT, Delhi, 218-224.

Wójcik M, Sugier P, Siebielec G (2014) Metal accumulation strategies in plants spontaneously inhabiting Zn-Pb waste deposits. Sci. Total Environ. 487, 313-322.

Zheljazkov VD, Nielsen NE (1996) Effect of heavy metals on peppermint and corn-mint. Plant soil 178, 59-66.
